# Supplementary material for: Higher trait mindfulness is associated with empathy but not with emotion recognition abilities
Source: R Soc Open Sci. 2020 Aug 5;7(8):192077. doi: 10.1098/rsos.192077 (PMC7481693; doi:10.1098/rsos.192077)
Supplement: Dataset supporting the article [file rsos192077supp1.pdf]

Supplemental Material

Higher Trait Mindfulness is Associated with Empathy But Not With Emotion Recognition Abilities

Ricardo Vilaverde, Ana Isabel Correia, and César F. Lima

Instituto Universitário de Lisboa (ISCTE-IUL)

Correspondence: cesar.lima@iscte-iul.pt

Table S1. *Descriptive Statistics for Each Emotion on the Emotion Recognition Tasks*

	M	*SD*	*Range*
Faces	.68	.11	.28 - .88
Anger	.69	.20	.08 - 1
Disgust	.66	.13	.26 - .92
Fear	.48	.21	.01 - .92
Happiness	.83	.15	.25 - 1
Sadness	.58	.18	.11 - 1
Surprise	.73	.15	.27 - 1
Neutrality	.79	.18	.19 - 1
Prosody	.62	.15	.14 - .87
Anger	.71	.22	.08 - 1
Disgust	.43	.29	0 - 1
Fear	.72	.23	0 - 1
Happiness	.61	.18	.19 - .93
Sadness	.70	.22	.04 - 1
Surprise	.53	.20	.08 - .92
Neutrality	.63	.19	.13 - 1

Table S2. *Correlations Among FFMQ Subscales*

Subscale	Describing	Acting with Awareness	Nonjudging	Nonreactivity
Observing	.22* (1.48)	.11(0.22)	-.06 (0.14)	.22* (1.56)
Describing	-	.45** (> 100)	.35** (81.73)	.17 (0.53)
Acting with Awareness	-	-	.34** (62.74)	.15 (0.40)
Nonjudging	-	-	-	.14 (0.33)

Note. * $p < .05$; ** $p < .01$; BF_{10} are indicated in parenthesis.

Table S3. *Correlations Between FFMQ Scores and Emotion Recognition Accuracy*

Emotion Recognition	FFMQ					
	Total	Observing	Describing	Acting with Awareness	Nonjudging	Nonreactivity
Total	-.10 (0.20)	.06 (0.14)	-.06 (0.14)	-.14 (0.33)	-.12 (0.25)	-.03 (0.13)
Faces (average)	-.10 (0.20)	-.01(0.12)	-.07 (0.16)	-.14 (0.33)	-.03 (0.13)	-.06 (0.14)
Anger	-.09 (0.18)	-.05 (0.14)	-.03 (0.13)	-.11 (0.22)	.00 (0.12)	-.09 (0.19)
Disgust	-.04 (0.13)	-.06 (0.15)	-.07 (0.16)	-.09 (0.18)	.03 (0.13)	.09 (0.18)
Fear	-.04 (0.13)	.10 (0.20)	.03 (0.13)	-.11 (0.23)	-.09 (0.19)	-.03 (0.13)
Happiness	-.20* (1.01)	-.10 (0.20)	-.16 (0.46)	-.07 (0.16)	-.15 (0.36)	-.14 (0.34)
Sadness	-.03 (0.13)	-.01 (0.12)	-.08 (0.17)	-.05 (0.13)	.06 (0.14)	-.03 (0.13)
Surprise	-.07 (0.16)	-.07 (0.15)	-.03 (0.13)	-.11 (0.23)	-.08 (0.17)	-.06 (0.15)
Neutrality	.03 (0.13)	.03 (0.13)	-.02 (0.12)	.00 (0.12)	.07 (0.15)	.00 (0.12)
Prosody (average)	-.07 (0.15)	.10 (0.19)	-.03 (0.13)	-.10 (0.19)	-.14 (0.35)	.00 (0.12)
Anger	.05 (0.14)	-.01 (0.12)	.09 (0.19)	.03 (0.13)	.02 (0.12)	-.01 (0.12)
Disgust	-.07 (0.16)	.14 (0.35)	-.03 (0.13)	-.17 (0.55)	-.16 (0.46)	.05 (0.13)
Fear	.02 (0.12)	.16 (0.47)	.04 (0.13)	.01 (0.12)	-.10 (0.20	-.04 (0.13)
Happiness	-.22* (1.39)	-.09 (0.18)	-.22* (1.49)	-.18 (0.71)	-.17 (0.57)	.08 (0.17)
Sadness	.01 (0.12)	.05 (0.14)	.06 (0.15)	-.05 (0.14)	-.02 (0.12)	-.03 (0.13)
Surprise	-.15 (0.40)	.06 (0.14)	-.12 (0.24)	-.10 (0.20)	-.21* (1.30)	-.06 (0.14)
Neutrality	.05 (0.14)	.10 (0.20)	.05 (0.13)	.02 (0.12)	.02 (0.12)	-.03 (0.13)

Note. * $p < .05$; BF_{10} are indicated in parenthesis.
